# Anti-inflammatory activities of arthropod peptides: a systematic review

**DOI:** 10.1590/1678-9199-JVATITD-2020-0152

**Published:** 2021-10-22

**Authors:** Ariane Teixeira dos Santos, Gabriela Silva Cruz, Gandhi Rádis Baptista

**Affiliations:** 1 Graduate Program in Pharmaceutical Sciences, School of Pharmacy, Dentistry and Nursing, Federal University of Ceará (UFC), Fortaleza, CE, Brazil.; 2 Laboratory of Biochemistry and Biotechnology, Institute for Marine Sciences, Federal University of Ceará (UFC), Fortaleza, CE, Brazil.

**Keywords:** Venom, Peptides, Anti-inflammatory, Arthropods

## Abstract

Peptides obtained from different animal species have gained importance recently due to research that aims to develop biopharmaceuticals with therapeutic potential. In this sense, arthropod venoms have drawn attention, not only because of their toxicity but mainly for the search for molecules with various bioactivities, including anti-inflammatory activity. The purpose of the present study is to gather data available in the literature on new peptides derived from arthropod species with anti-inflammatory potential. This systematic review followed the Preferred Reporting Items for Systematic Reviews and Meta-Analysis (PRISMA) guidelines. Studies on peptides from arthropods that display anti-inflammatory activity were retrieved from PubMed, Scopus, Web of Science, and Google Scholar databases. The bibliographic research started in 2020 and searched papers without a limit on the publication date. The articles were analyzed using a search string containing the following terms: “Peptides” and “Anti-inflammatory”, in combinations such as “Ant”, “Bee”, “Wasp”, “Crab”, “Shrimp”, “Scorpion”, “Spider”, “Tick” and “Centipedes”. Besides, a search was carried out in the databases with the terms: “Peptides”, “Antitumor”, or “Anticancer”, and “Arthropods”. Articles that met the inclusion and exclusion criteria totalized 171, and these served for data extraction. Additionally, the present review included anti-inflammatory peptides with anticancer properties. Peptides with confirmed anti-inflammatory activity were from insects (ants, bees, and wasps), crustaceans (shrimp and crabs), arachnids (scorpions, spiders, and ticks), and centipedes. These arthropod peptides act mainly by decreasing pro-inflammatory cytokines as analyzed *in vitro* and *in vivo*. Some showed significant antineoplastic activity, working in essential cellular pathways against malignant neoplasms.

## Background

The use of enzymes and polypeptides for medicinal purposes has attracted considerable interest due to their high specificity and selectivity. They are also less likely to interfere with cellular processes that are not the aimed therapeutic targets. Protein drugs are composed of bioactive polypeptides with significant therapeutic potential [1]. Although animal venoms have toxic effects, they are extensively studied to find pharmacologically active molecules [2]. A known example of an isolated venom component that served as a template for developing the anti-hypertensive drug captopril belongs to the bradykinin-potentiating peptide (BPP) family found in the venom of *Bothrops jararaca* [3].

Arthropods comprise one of the largest groups of animals on Earth, with diverse species being venomous. These species contain complex mixtures of components in their venoms with various families of toxins that exert numerous biological effects on target organisms and systems, testified by a growing number of reported studies available in public databases. This kind of natural chemical and peptide library provides excellent potential for discovering new compounds and activities for alternative or adjuvant therapies based on the mimetic modulation of pharmacological activities of endogenous (poly)peptides in the body [4-6]. More than 400 toxins from various animals have activities reported in the literature, and around 3400 reported proteins are from arthropods [7].

Natural products comprise an essential source of bioactive substances, and they have contributed significantly to the manufacture of old and new drugs for diverse therapeutic purposes. In recent years, of all the molecules approved by the U. S. Food and Drug Administration (FDA), a third of them are natural products and derivatives from mammals and microbes [8]. However, arthropod venoms as sources of new pharmaceutically functional molecules are yet to be deeply explored [9]. Many arthropod venom peptides represent an opportunity by which venom components could be converted into “pharmaceutical gold” [10,11,12]. The production of a drug derived from venoms also includes the characterization of synthetic or recombinant peptide forms. Examples include peptides capable of modulating and/or regulating pain [13]. 

This review presents examples of peptides from various arthropod species, mainly focused on biologically active peptides found in arthropod venom with anti-inflammatory potential.

## Methods

### Investigation plan

This systematic review followed the Preferred Reporting Items for Systematic Reviews and Meta-Analysis (PRISMA) guidelines [14]. The search of published articles on the topic of arthropod-derived peptides with anti-inflammatory activity was through PubMed, Scopus, Web of Science, and Google Scholar electronic databases. The bibliographic retrieval started in August 2020 and finished in March 2021. The search did not limit the date of publication. The publications were analyzed using a search string containing terms: “Peptides” and “Anti-inflammatory”, in combinations such as “Ant”, “Bee”, “Wasp”, “Crab”, “Shrimp”, “Scorpion”, “Spider”, “Tick” and “Centipede”. In addition, a search was carried out in the databases with the terms: “Peptides”, “Antitumor”, or “Anticancer”, and “Arthropods”.

### Selection of the literature

The studies were selected by the coauthors’ ATS and GSC through Mendeley software (version 1803, 2020) and verified by GRB, ensuring the review work’s inclusion. The selected literature adhered to the following criteria: full research articles that have been conducted *in vitro* or *in vivo* experimental studies and evaluated the anti-inflammatory effects of peptides derived from arthropod venoms or their crude extract. Besides, included in this review are ethnopharmacological data related to the topic covered. The criteria used to exclude studies were: repeated articles, editorials, letters to the editor, thesis, dissertations, reports, and articles that are out of the scope of this review.

### Data collection

According to the required criteria, the studies selected for inclusion in this systematic review were chosen by the authors’ ATS and GSC. The information collected from the literature contains the following information: authors, affiliation, year of publication, applied methodology, characterized compound, and main results.

## Results

**Figure 1.**
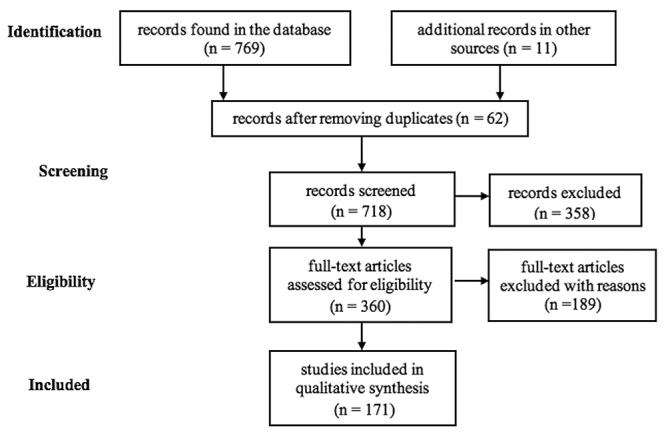
PRISMA flowchart showing the research design process of the study.

After searching the databases, 171 original and review articles were selected out of 769 published papers and utilized to prepare the current review. The flow diagram (Figure 1) depicts the details of the selection process in the databases. Also, general information was obtained, referring to the article’s title, authorship, and publication year.

Reading the material in its entirety made it possible to identify specific information about the animal species involved in the study, the peptide structure identified as a potential anti-inflammatory agent, and the anti-inflammatory activity described more precisely. Table 1 summarizes the collection of this information.

**Table 1.** Examples of peptides from the Uniprot database with anti-inflammatory activies.

Animal (Source)	Peptide	Access number	Activity as inflammatory mediator	Ref.
**Insect**				
*Pseudomyrmex triplarinus*	Pseudomyrmecitoxin-Pt1 subunit LS1	P0DSL7	Antidematogenic effect	[19-21]
	Pseudomyrmecitoxin-Pt1 subunit SS3	P0DSM1		
	Pseudomyrmecitoxin-Pt1 subunit LS2	P0DSL8		
	Pseudomyrmecitoxin-Pt1 subunit SS2	P0SDM0		
	U1-pseudomyrmecitoxin-Pt1 subunit SS1	P0DSL9		
*Paraponera clavata*	Delta-paraponeritoxin-Pc1a	P41736	Edema reduction, antinociceptive	[22]
*Dinoponera quadríceps*	Venom peptides (Extract)	C0HJK0	Suppression of inflammatory mediators	[23-26]
*Brachyponera sennaarensisare*	Venom peptides (Extract)	-	Regulate the expression of MHC-II, CD80 y CD-86, IFN-γ and IL-17	[28,29]
*Pachycondyla sennaarensis*	Venom peptides (Extract)	-	Regulate NF-kB, kinase IkB, TNF-α and Fas	[39]
*Apis melífera*	Venom peptides (Extract)	-	Reduction the levels of inflammatory mediators	[40-46]
	Phospolipase A2	P00630	Reduction of apoptotic levels mediated by Bcl-2 and Bcl-xL	[55-57]
	Melittin	P01501	Inactivation of NF-kB	[58-67]
	Apamin	P01500	Suppression Th2-related chemokines/Regulation the activation of the NF-kB, STATS 1 and 2 pathways	[69-71]
	Adolapin	-	Reduction of paw edema, the levels of prostaglandins, cyclooxygenase 2, in addition to inhibiting PLA2 activity	[72-74]
**Crustacean**				
*Protopolybia exígua*	Mastoporan-1	P69034	Inhibition Toll-like receptor 4 (TLR4) mRNA, suppressionTNF-α and interleukin-6 (IL-6)	[80]
*Nasonia vitripennis*	Venom peptides (Extract)	-	ReductionIL-1β, IL-6 and NF-kB	[82,83]
*Vespa magnificca*	-	P0CH47	Inhibition of the NF-kB pathway	[84]
*Limulus polyphemus*	Anti-lipopolysaccharide factor	P07086	Immunomodulatory activity	[67,68]
*Penaeus monodon*	Anti-lipopolysaccharide factor	B1NMC7	Disruption of the mitogen-activated protein (MAP) pathway by regulating and reducing the release of pro-inflammatory cytokines	[90-91]
*Portunus trituberculatus*	Anti-lipopolysaccharide factor	C0KJQ4	Immunomodulatory activity	[98-102]
	Anti-lipopolysaccharide factor isoform 4	H9MYY2		
	Anti-lipopolysaccharide factor isoform 5	-		
	Antilipopolysaccharide factor isoform 8	-		
*Scylla paramamosain*	Catalase	D0EVW7	Antioxidant potential	[105,106]
*Scylla serrata*	Anti-lipopolysaccharide factor	B5TTX7		
*Charybdis natator*	Crab leg	-	Modulating the NF-kB pathway	[107]
**Arachnid**				
*Titius obscurus*	Toxin To3	P60213	Suppression of TNF-α and IL-1β	[112]
	Toxin To4	P60215		
*Tityus stigmurus*	Hyaluronidase	P0C8X3	Reduction the migration of leukocytes and TNF-α release	[113]
*Tityus serrulatus*	Antimicrobial peptide TsAP-2	S6D3A7		
*Mesobuthus martensii*	Makatoxin-1	P56569	Reduction the production of inflammatory mediators such as nitric oxide (NO), TNF -α, IL-6 and IL-1β	[116]
	Potassium channel toxin alpha-KTx 3.6	Q9NII7	Suppress cytokine secretion	[119, 121, 122]
*Heterometrus laoticus*	Hetlaxin	C0HJN0	Act on Kv1.3 potassium channel	[123]
*Heterosodra maculata*	Delta-theraphotoxin-Hm1a	P60992	To control thehypersensitivity and chronic visceral pain	[125]
*Phlogiellus* sp.	Phlotoxin 1	P0DM14	Antinociceptive activity	[128]
*Phoneutria nigriventer*	Phα1β	P81789	Anti-inflammatory and antinociceptive	[132-135]
	Tx3-3	-		
	PnTx4			
	PhKv			
*Pardosa astrigera*	Lycotoxin-Pa4a	-	Suppresses nitric oxide, nitric oxide-induced synthase (iNOS), IL-1β, TNF-α	[136]
*Ornithodoros savignyi*	OsDef2	-	Inhibits the production of TNF-α and NO-induced	[139]
*Hyalomma asiaticum*	Hyalomin-A1	-	Inhibits the secretion of pro-inflammatory cytokines and increasing the secretion of IL-10	[140,141]
	Hyalomin-B1			
*Rhipicephalus sanguineus*	Evasin-1	P0C8E7	Inhibits cell of chemokines CCL3, CCL3L1, and CCL4 and CCL5	[142]
	Evasin 3	P0C8E8		
	Evasin 4	P0C8E9		
*Amblyomma variegatum*	Amphiregulin	-	Inhibitis the secretion of TNF-α, IL-1, IL-8, and IFN-γ	[143]
**Chilopod**				
*Scolopendra subspinipes*	Formyl peptide receptor 2	-	Inhibitis the release of pro-inflammatory cytokines and the recruitment of neutrophils in the joint	[147]
	Scolopendrasin IX	-	down-regulate the expression of pro-inflammatory mediators such as TNF-α and IL-6	

Source: Uniprot database.

### Insect peptides

*Ants*

Insects possess a multitude of unexplored toxins with presumed potent biological activities. For instance, ants (Insecta class, Hymenoptera order, Formicidae family) are mostly venomous and express several types of peptides in their venoms, therefore emerging as an essential source of bioactive peptides [15]. Not so long ago, investigating the biological effects of isolated peptide toxins from insects was hampered by the size of these majorly tiny animals. With the advent of omics technology, the discovery and characterization of novel peptides progressed [16]. Initial studies aimed to unveil a way to alleviate the secondary effects caused by these animals’ bites, with ants belonging to the genera Solenopsis, Pachycondyla spp, and Myrmecia the most studied [17, 18]. In crude and isolated forms, the characterization and verification of several bioactive peptides from the venom of *Pseudomyrmex* species, such as the mirmexin peptide, proved to have a potent antidematogenic activity [19-21]. As observed *in vivo,* poneratoxin, a 25-residue peptide from the bullet ant *Paraponera clavate*, and some Formicidae peptides, can reduce edema, besides their antinociceptive activity [22]. In the context of ethnopharmacology, there are reports about the topical use of macerated giant ants *Dinopera quadriceps* for the treatment of back pain and rheumatic cases [23]. These studies have shown that the crude extracts reduced paw edema, leukocyte migration, malonaldehyde, and nitrite content, ameliorating acute peritonitis *in vivo* and *in vitro*. This extract contained modulator molecules of cellular oxidant/antioxidant mechanisms involved in acute inflammation elicited by zymosan, but more specific mechanisms of action have not been described [24,25]. The crude venom of this species has the potential to reduce nociception and interleukin-1β (IL-1β), which suggests that it suppresses inflammatory mediators such as cyclooxygenase-2 (COX-2) and prostaglandin-2 (PGE-2) involved with pain [26,27]. The *Brachyponera sennaarensisare* (Samsum ant) ant-derived toxins modulate not only pain but also the immune response. The *B. sennaarensisare* toxins regulate the expression of MHC-II, CD80, and CD-86, as well as interferon- γ (IFN-γ) and interleukin-17 (IL-17), mediators that are involved in various chronic pathologies and cancer as demonstrated after in vivo tests [28]. Furthermore, these peptides can regulate the nuclear factor kappa B (NF-kB), kinase IkB upward, and suppress nuclear transcription factor-α (TNF-α) and the cell surface death receptor (Fas), although the mechanism involved in anti-inflammatory activity has not been fully elucidated [29,30].

*Bees*

Bees are part of the class Insecta, order Hymenoptera, family Apoidea, and clade Anthophilia. In Brazil, bee venom is commonly found and consists of various bioactive agents that induce allergic reactions when injected into the human body [31]. However, its use for medicinal purposes was documented approximately 6,000 years ago [32]. Bee venom therapy (BV) is a form of medicine native to ancient Greece and China [33]. In recent years, bee-based therapy has become a new treatment option. An increasing body of scientific evidence has demonstrated the therapeutic potential of bee venom [34]. In traditional medicine in Asia, BV was used in conjunction with acupuncture to treat some anti-inflammatory diseases. Furthermore, combination therapy can reduce inflammation in amyotrophic lateral sclerosis (ALS) due to the disease’s side effects on the liver, kidney, and spleen [35]. Combination acupuncture and BV therapy (i.e., *Apis mellifera* crude venom) were also favorable to treat respiratory inflammation accompanied by leukocyte, myeloperoxidase (MPO), and IL-1 suppression, using a carrageenan-induced pleurisy mouse model [36]. 

The inflammation suppression mechanism of *European honey bee Apis mellifera* BV, observed in previous studies with animal models, also reduces the formation of atherosclerotic lesions by decreasing the intercellular adhesion molecule 1 (ICAM-1), vascular adhesion molecule- 1 (VCAM -1), and transforming growth factor-β1 (TGF-β1) [37]. Furthermore, the reduction of inflammation induced by apitoxins - a venom bee peptide (*A. mellifera*) component, is due to the decrease in apoptotic levels mediated by Bcl-2 and Bcl-xL and activating BCL2-associated X protein (Bax) and caspase-3 [38]. The application of bee venoms (*A. mellifera*) extends to reduce inflammatory lesions caused by the bacteria *Propionibacterium acnes* through decreasing TNF-α, interleukin-8 (IL-8), and IFN-γ, while also blocking the expression of Toll-like receptor 2 (TLR2) in human keratinocytes and monocytes [39].

Based on previous studies, bee venom toxins from *A. mellifera* and *A. cerana indica* act by regulating NF-kB signaling; the antiarthritic effect has been explored to reduce the levels of inflammatory mediators directly involved in the pathophysiology of rheumatoid arthritis, similarly to standard drugs such as methotrexate [40-46]. The compound bee venom’s potential extends to reducing pain, acting as an antinociceptive agent by modulating the α2-adrenergic receptor and cyclooxygenase-2, accompanied by suppressing edema [47-51]. BV has a broad spectrum of activities. Its effects are not limited only to joint diseases and respiratory diseases, promoting an improvement in the allergic condition by suppressing inflammatory cytokines when tested in an allergic chronic rhinosinusitis mouse model [52].

Bee venom is a complex mixture that includes proteins and peptides such as melittin, apamin, phospholipase A2, phospholipase B, hyaluronidase, phosphatase, α-glucosidase, MDC peptide, and adolapin, among other minor components [53,54]. Secretory phospholipase A2 (PLA2- *Apis mellifera*), a prototype enzyme in bee venom, hydrolyze fatty acids while also having a role in protecting liver damage by producing anti-inflammatory cytokines in mice and reducing neuroinflammation by reducing phosphorylation of STAT3 and inflammatory mediators, including p-STAT3 [55,56]. Bee venom phospholipase A2 ameliorates amyloidogenesis and neuroinflammation by inhibiting signal transducer and activating the transcription-3 pathway in Tg2576 mice [57].

Melittin (*Apis mellifera*), one of the main peptides in bee venom, comprises 26 amino acid residues with an overall amphipathic character. Administration in high doses of this apitoxin can trigger an allergic reaction, causing local itching and pain. In low doses, it may have an anti-inflammatory role by inhibiting the enzymatic activity of PLA2. Synthetic melittin inhibited the enzymatic activity of secretory phospholipase A2 (PLA2) from various sources, including bee and snake venoms, bovine pancreas, and synovial fluid from rheumatoid arthritis patients. Based on melittin’s hydrophobic nature and its capacity to bind to PLA2, melittin could act as a carrier for PLA2 to translocate it to the membrane. Melittin inhibits the bee venom PLA2 noncompetitively by binding to the enzyme domain other than the catalytic site. [58]. The protective effect of melittin on inflammation and apoptosis was also observed in acute liver failure; the treatment with melittin attenuated the increase of inflammatory cytokines and significantly inhibited caspase expression Bax protein levels, as well as cytochrome *c* release in vivo [59,60].

Moreover, the JNK-dependent inactivation of NF-kB caused by melittin may prevent the release of inflammatory mediators involved in oxidative stress and the generation of pain [61]. Melittin-induced inhibition of this signaling pathway, which included the ERK and AkT cascade, and suppression of the inflammatory mediators upregulated in periodontitis, a chronic inflammatory disease, was observed in *P. gingivalis* LPS-stimulated human keratinocytes [62]. Melittin also reduced the release of pro-inflammatory cytokines by monocytes after contact with *P. acnes*. It is also an effective agent that prevents liver fibrosis by inhibiting inflammation by interrupting the NF-κB signaling pathway [63-64]. Moreover, melittin modulated inflammation, having better activity and less toxicity when associated with glutathione S-transferase while *in vitro*. When using doses that exceed the toxic concentration, it still retains its inflammatory properties [65]. A study reports its beneficial effect in treating inflammatory diseases, including skin inflammation, neuroinflammation, atherosclerosis, arthritis, and liver inflammation [66].

Apamine is another toxin that constitutes bee venom. It is an 18 amino acid-residue neurotoxic peptide. Despite its neurotoxicity, apamine helps treat Parkinson’s disease or learning deficits [67]. Moreover, apamine, as an anti-inflammatory peptide, reduced the paw’s volume and the haptoglobin and seromucoid contents *in vivo* [68,69]. This bee venom peptide was efficient in treating atopic dermatitis. The Apamin inhibits TNF-α- and IFN-γ-induced inflammatory cytokines and chemokines via suppressions of NF-κB signaling pathway and STAT in human keratinocytes [70]. Apamine showed anti-inflammatory effects in mice with gouty arthritis by inhibiting pro-inflammatory cytokine production and inflammasome formation [71].

 Adolapin, from *A. mellifera* venom, is another bee venom peptide with potent anti-inflammatory effects but not as well studied as melittin. It reduces the edema of the paw in mice, the levels of prostaglandins, cyclooxygenase 2, in addition to inhibiting PLA2 activity. The anti-inflammatory activity of adolapin is evident in carrageenin models, prostaglandin, rat hind paw edemas, and adjuvant polyarthritis. The adolapin effects are presumably due to its capacity to inhibit the prostaglandin synthase system, following a biphasic dose-response relationship. Likely, among the central mechanisms, one involved an analgesic action of adolapin [72]. Peptide 401 (mast cell degranulating peptide - MCD peptide), with 22 amino acid residues, considered a potent degranulation factor for bee venom mast cells, substantially inhibited the edema caused in rats and attenuated the inflammatory process at the affected site [73,74].

*Wasps*

Like bees, wasps (Insecta, Hymenoptera, Apocrita) have complex mixtures of toxins in their venoms and have attracted interest as a potential arthropod source of bioactive substances. Wasps belong to the family Vespidae, and members include the genus Dolichovespula (wasp), Vespula (yellow wasps), and Polistes (paper wasps) [75]. When injected, the wasp toxins trigger local adverse effects such as pain, edema, erythema, and immune reactions such as anaphylaxis [76,77]. In general, wasps’ venom comprises a cocktail of hydrophobic peptides, including amines, peptides, enzymes, allergens, and toxins [78,79]. For example, mastoparan is an amphipathic, 14-amino acid residue, and it was the first peptide isolated from wasps. This toxin is found in the genera *Vespa, Parapolybia, Protonectarina, Polistes, Protopolybia* [80].

Like bee venom, wasps’ venoms have a considerable anti-inflammatory effect, shown in*in vitro*studies. These contain toxins that have the potential to inhibit Toll-like receptor 4 (TLR4) mRNA expression, in addition to suppressing TNF-α and interleukin-6 (IL-6) [81]. Although crude venoms contain several toxins that can trigger a toxic reaction, wasp venoms have powerful anti-inflammatory complexes, as is the case of the crude venom of the wasp *Nasonia vitripennis* (jewel wasp). The *N. vitripennis* crude venom reduced the expression of inflammatory cytokines directly involved in inflammatory processes mediated by IL-1β, IL-6, and NF-kB [82,83]. In an arthritis model, crude wasp venoms caused the inhibition of the NF-kB pathway. Likewise, *Vespa magnifica* (murder hornet) and other wasp species’ crude venoms suppressed the expression of mediators involved in hyperalgesia and rheumatoid arthritis [84-88].

A study dealing with *Vespa tropica* (Greater banded hornet) showed that crude venom significantly reduced oxidative stress and the mouse microglial cell line activation, previously stimulated by LPS. Moreover, the peptides purified from the crude venom exhibited potential anti-inflammatory properties, targeting the p38 and MAPK pathways, causing the suppression of NF-κB phosphorylation in LPS-stimulated cells [89].

### Crustacean peptides

*Prawns/shrimps*

Despite not being poisonous, shrimps (Crustacea, Malacostraca, Decapoda) were included here because they do not have an adaptive immune system and therefore rely on their innate immunity bioactive peptide components to deter invading pathogens. Antimicrobial peptides (AMP) are responsible for the immediate host response against invading bacteria, fungi, parasites, and, in some cases, they connect the innate and the adaptive immune response by modulating the expression and release of cytokines. The primary AMPs found in shrimp are grouped into three families of cationic peptides, namely, penaeidins, crustines, and anti-lipopolysaccharide factor (ALF) [90]. The ALF, firstly discovered in the horseshoe crab (*Limulus polyphemus*), was followed by the identification in other crustacean species, like in the black tiger prawn*Penaeus monodon*, being designated SALF (Shrimp Anti-Factor Lipopolysaccharide) [90,91]. It is a precursor molecule with a signal sequence of 22 to 28 residues, followed by a mature peptide that contains two conserved cysteine residues. ALF’s functional domain is named lipopolysaccharide-binding domain (LPS-BD) and contains the primary amino acids involved in recognizing and binding LPS and other components of Gram-positive bacteria and fungi [92].

*P. monodon* shrimp contain eleven ALF isoforms distributed in seven groups (Group A to Group G). Likewise, these isoforms can be found in the shrimp species*Farfantepenaeus aztecus* (brown shrimp),*L. vannamei* (pacific white shrimp or king prawn), and*Marsupenaeus japonicus*(known as the kuruma shrimp, kuruma prawn, or Japanese tiger prawn) [91,92]. LPS is an endotoxin present in the outer cell membrane of Gram-negative bacteria. When in contact with the host, it binds to pathogen recognition receptors that recognize this pathogen-associated molecular pattern (PAMP) and activates the signaling pathways that initiate the inflammatory process [93]. Recent studies show that SALF, besides antimicrobial activity, plays an essential role in neutralizing LPS and preventing its binding to the TLR-4 type Toll-like receptor (TLR). This peptide could inhibit or reduce the inflammatory response, disrupting the mitogen-activated protein (MAP) pathway by regulating and reducing the release of pro-inflammatory cytokines after *in vitro* tests with different cell lines [93-96].

Among studies about the efficacy of SALF as an anti-inflammatory agent, the effects of *Penaeus monodon* (giant tiger prawn) SALF on the production and release of tumor necrosis factor (TNF) were reported. This peptide showed suppression of inflammation in a dose-dependent manner in LPS-stimulated cervical cancer HeLa cells. Although the results have been promising, the mechanism involved in anti-inflammatory activity has not been fully elucidated [93]. The SALF peptides’ protective role includes an anti-inflammatory effect in response to LPS, as observed in cervical cancer epithelial cells (HELA cells). SALF fragments inhibited inflammatory cytokines production, including TNF, interleukin IL-1 β, IL-6, IL-1, and monocyte chemoactive protein (MCP-1). SALF also suppressed IL-6, IL-8, IL-1, and MPC-1e mRNA levels and regulated vaginal epithelial cell immune responses through MAPK (mitogen-activated protein kinases) and NF-κβ (nuclear factor kappa B) pathways [93].

In addition to the SALF response to bacterial LPS, this peptide modulates the inflammatory responses provoked by the protozoan *Trichomonas vaginalis*, an etiological agent of Trichomoniasis that affects the cervicovaginal mucosa. When vaginal cells were subjected to stimulation by*T. vaginalis*, SALF inhibited the release of pro-inflammatory cytokines such as TNF-α, IL-6, IL-8, and MCP-1 through the MAPK pathways and NF-κβ [96]. These reports exemplify the promising profile of SALF as an anti-inflammatory agent.

*Crabs*

In recent years, marine organisms have attracted great interest due to their unique constituents with diverse bioactivities. These animals have hemolymph with potent antimicrobial peptides essential for their innate immunity. These peptides are valuable for biomedical applications [97]. Crabs (Crustacea, Malacostraca, Decapoda, Pleocyemata) have been investigated for the peptides’ antimicrobial activity and their immunomodulatory effects. Purified peptides from various species of crabs such as LALF (The Atlantic horseshoe crab*-Limulus polyphemus*), M-ALF (kuruma shrimp-*Marsupenaeus Japonicus*), PtALF, PtALF4, PtALF5, and PtALF8 (horse crab-*Portunus trituberculatus*) showed an anti-lipopolysaccharide activity [98-103]. In another example, the β-1,3-glucan binding protein (β-GPB) from the rice paddy crab*Paratelphusa hydrodromus* can trigger an immune response against external aggressors. Additionally, β-GPB also exerts an antioxidant effect, reducing DPPH radicals, in a model of restraining the albumin’s denaturation [104]. Regarding the antioxidant enzymatic profile, enzymes purified from distinct crab species showed an effective antioxidant potential by increasing the activity of superoxide dismutase (SOD) and catalase (CAT) [105,106]. Moreover, crab-derived peptides can restrain the inflammatory process by reducing inflammatory mediators’ levels and modulating the NF-kB pathway, implicated in various inflammatory diseases [107]. Besides their role as an anti-inflammatory substance, these crustacean-derived peptides can exert antinociceptive effects, consequently playing a role in pain control as potent COX-2 reducers *in vitro* [108].

### Arachnida peptides

*Scorpions*

Venom peptides from scorpion (Chelicerata, Arachnida, Scorpiones) distribute into two main groups: DBPs (disulfide-bridged peptides) and NDBPs (non-disulfide-bridged peptides). DBPs generally target ion channels. Most scorpion DBPs contain three to four disulfide bridges and interact with the Na+, K+, Ca2+, and Cl− channels. In comparison, the NDBP peptides are less abundantly distribute among scorpion venoms and exhibit multiple activities, such as insecticide, antiviral, antimicrobial, hemolytic, antiproliferative, bradykinin-enhancing, and immunomodulatory [109,110]. 

Dias and collaborators [111] analyzed 320 non-disulfide bond-containing peptides, of which 27 had their sequences assigned. Among them, thirteen peptides constituting novel toxins in*Tityus obscurus* venom (Amazonian black scorpion). As examples, ToAP3 (FIGMIPGLIGGLISAIK-NH2) and ToAP4 (FFSLIPSLIGGLVSAIK-NH2) NDBPs exerted their effect on immunomodulation and suppression of inflammatory mediators, such as TNF-α and IL-1β. Furthermore, ToAP3 and ToAP4 were associated with the modulation of antigen presentation. They reduced TNF-α and IL-1β at transcriptional and translational levels in bone marrow-derived macrophages (BMDM) and dendritic cells (BMDC). The reduction of TNF-α secretion before LPS- inflammatory stimuli is associated with peptide interaction with TLR-4. ToAP4 increased MHC-II expression in BMDC, while ToAP3 decreased co-stimulatory molecules such as CD80 and CD86 [112]. Stigmurin, a cationic peptide from the scorpion *Tityus stigmurus* venom (scorpion from the family Buthidae found in Brazil) and TsAP-2 from the scorpion *Tityus serrulatus* venom (Brazilian yellow scorpion) both reduced the migration of leukocytes and TNF-α release, reducing the inflammatory process. Additionally, the fractions extracted from their respective crude venoms could modulate the expression of the cytokines IL-4, IL-6, IL-13, and IL-13, which are pro and anti-inflammatory [113].

The peptide Ts14 from *T. serrulatus* modulates critical events occurring in the fibrovascular tissue, i.e., it causes neovascularization, inflammatory cell recruitment, and extracellular matrix deposition induced by polyether-polyurethane sponge implants in mice. Consequently, Ts14 has therapeutic potential in wound healing and ischemic and inflammatory conditions. Furthermore, Ts14 reduced TNF-α levels and neutrophil infiltration, although stimulated macrophage infiltration into implants, as determined by myeloperoxidase (MPO) and N-acetyl-β-d-glucosaminidase (NAG) enzyme activities, respectively [114]. BotAF is a peptide derived from *Buthus occitanus tunetanus* (common yellow scorpion), another yellow scorpion species that comprises a long chain of 64 amino acid residues, with potent analgesic activity in rodents [115]. From the Chinese scorpion*Mesobuthus martensii* (Chinese scorpion), 35 scorpion oligopeptides (CMOs) were studied. Specifically, the peptide CMO-1 suppressed inflammation by reducing the production of inflammatory mediators such as nitric oxide (NO), TNF -α, IL-6, and IL-1β in RAW264.7 macrophages cells. Moreover, CMO-1 inhibited the degradation of IkBα and the nuclear translocation of p65. It also suppressed NF-kβ activation and inhibited MAPK phosphorylation of ERK, JNK, and p38 [116]. The venom of another species of *Mesobuthus* (*Mesobuthus eupeus*- lesser Asian scorpion*,*the lesser Asian scorpion, or the mottled scorpion) was effective in treating CFA-induced arthritis, in which the edema reduction correlated with the reduction of arthritis [117]. 

Sc20 from the venom of *Scorpiops tibetanus* is also a potent anti-inflammatory and immunosuppressor. This peptide modulated two important pro-inflammatory factors: the secretion of TNF-α and IFN-γ, displaying a positive effect in delayed hypersensitivity. Similar peptide St20, the first disulfide-bridged toxin peptide from the scorpion *S. tibetanus*, showed immunosuppressive and anti-inflammatory activities, suggesting that it may be a novel source of venom peptides to treat human disease [118].

The voltage-gated Kv1.3 channel, expressed in memory-efficient T cells, is presently a recognized targeted drug for treating various autoimmune diseases. Scorpion venom possesses Kv1.3 channel peptide blockers that suppress cytokine secretion and alleviate disease in animal models of T-cell-mediated autoimmune diseases [119]. Thus, to improve the selectivity and activity of these scorpion venom peptides directed at regulating Kv1.3 potassium channels are currently undertaken. A remarkable example is the study of the scorpion toxin BmKTX, isolated from *M. martensii* [120]. Recently, BmKTX analogs such as ADWX-1, BmKTX-D33H, BmKTX-19, and BmKTX-196 demonstrated specific inhibition of the Kv1.3 channel. Most venom-derived peptides have not evolved to target specific mammalian receptors of therapeutic interest; therefore, preparing peptide analogs with higher potency toward specific targets is customary [119,120,121]. The Vm24 scorpion toxin also showed similar activity to the venom-peptides above, which are blockers of Kv1.3 channels, acting without affecting the T cells’ viability and inhibiting the activation of CD25 and CD40L, as well as the cytokine secretion of pro-inflammatory IFN-γ and TNF [122].

Hetlaxin (ISCTGSKQCYDPCKKKTGCPNAKCMNKSCKCYGC) is a DBPs, belonging to the scorpion alpha-toxin family, isolated from the *Heterometrus laoticus* venom (Vietnam forest scorpion), which possesses a high affinity to the Kv1.3 potassium channel. This isolated *H. laoticus* venom peptide exerted an anti-inflammatory effect similar or slightly superior to ketoprofen [123].

*Spiders*

Spiders (Chelicerata, Arachnida, Araneae) comprise one of the oldest living animals on Earth that surged approximately 300 million years ago and comprise the most significant number of living species (> 40,000) [124]. As in other arthropods, inoculation of their venom causes local discomfort, such as edema, and more severe deleterious effects, like ulcerations, acute renal failure, and even death in the worse cases [125,126]. Although arachnids venoms are harmfully toxic to humans, some venom peptides have beneficial bioactivities applicable to biomedicine. In general, arthropod-derived venom’s biochemical targets are excitable neuronal receptors; these include ion channels like voltage-gated sodium channels (Nav) found in neurons, which allow the modulating of pain. Spider peptides that modulate such pharmacological targets serve as molecular templates for the development of analgesic drugs. For example, the Hm1a peptide purified from the venom of *Heterosodra maculate* (togo starburst baboon spider) can control the hypersensitivity in chronic visceral pain [127].

Phlotoxin 1 (Ph1Tx1) is a 34-residue toxin purified from *Phlogiellus spider* venom, a promising antinociceptive peptide with a high affinity for Pav [128]. The crude venom of *Phoneutria nigriventer* (armed spiders), besides its antineoplastic activity, can suppress the IFN-γ release and increase the expression of the anti-inflammatory cytokine IL-10. Phα1β, a peptide purified from the venom of *P. nigriventer*, has a significant role in the control of the CFA-induced chronic arthritis model. The* *Phα1β suppressed the inflammatory agent’s side effects while the antinociceptive role acted as the antagonist of the TRAP1 channel [129-131]. Furthermore, other peptides such as Tx3-3, PnTx4, PhKv, and PhTx3-5 from the *P. nigriventer* venom have important antinociceptive properties as observed in the animal neuropathic inflammatory pain model [132-135]. Lycotoxin-Pa4a peptide from *Pardosa astrigera* venom displays immunomodulatory activity by increasing the expression of IL-10 and suppressing pro-inflammatory mediators such as nitric oxide, nitric oxide-induced synthase (iNOS), IL-1β, TNF-α, in addition to reducing COX-2. *In vitro* studies with an LPS-stimulated model demonstrated that this peptide could act as a potential antinociceptive modulator [136].

*Ticks*

Ticks are hematophagous arthropods that rely only on the innate defense to protect themselves against invading microorganisms. Biologically active molecules are also necessary to keep blood fluid during feeding and eliminate the host’s defense mechanisms, such as vasoconstriction, forming a hemostatic plug, activating the coagulation cascade, and initiating inflammatory responses that lead to wound healing and tissue remodeling. Thus, some bioactive molecules have anticoagulant, antiplatelet, vasodilatory, anti-inflammatory, and immunomodulatory activity and are crucial to overcoming the host’s hemostatic and immunological responses, allowing ticks to feed and develop [137].

*Ornithodoros savignyi* (sand tampan, African-eyed tampan, or Kalahari sand tampan) is a tick that parasites cattle and is endemic in arid and semi-arid regions of the African continent. This tick species express antimicrobial peptides (defensins) constitutively in various tissues at low levels and inductively during blood-feeding or in response to bacterial challenge. Defensins are cationic molecules with molecular masses of approximately 4 kDa containing cysteine residues forming three disulfide bonds [138]. Studies on *O. savignyi* resulted in the cloning and sequencing of defensin isoforms, OsDef1 and OsDef2, derived from the terminal carboxy region. Due to the bactericidal activity isoform 2, this peptide served as a model for the synthesis of the peptide Os (KGIRGYKGGYCKGAFKQTCKCY) and its analog Os-C (KGIRGYKGGY- KGAFKQT- K-Y), with 22 and 19 residues of amino acids, respectively [139]. Os peptides’ mechanisms of action in bacterial cells’ membrane involve their penetration into the cell and action on intracellular targets. As a result of these findings, Malan et al. [139] evaluated these peptides’ effects in inflammatory conditions resulting from gram-negative bacteria infection. Thus, Os and Os-C’s showed anti-inflammatory properties on Raw 264.7 macrophages stimulated by LPS and IFN-γ *in vitro*. Both peptides inhibited the production of TNF-α and NO-induced by LPS in RAW 264.7 cells without appreciable cytotoxic effects. In addition to anti-endotoxin activity and anti-inflammatory properties, Os eliminated NO directly, and both Os and Os-C peptides exhibited antioxidant activity, which together can reduce oxidative stress associated with inflammation [139].

Wu et al. identified two families of immunoregulatory peptides, hyalomin-A1 and hyalomin-B1, from the salivary glands of the *Hyalomma asiaticum* tick. The amino acid sequences of hyalomin-A1 and B1 correspond to the sequences QTPRTIGPPYT and TLRTTTGYWTTVEKGNGTTPAANSTEKGNRPYGR, respectively. Hyalomin-A1 and B1 act as immunoregulators, inhibiting the secretion of pro-inflammatory cytokines induced by LPS in vitro and increasing immunosuppressive cytokine, IL-10 [140]. Both hyalomin-A1 and B1 could quickly eliminate oxidants in a few seconds. Such antioxidant activities can contribute to immunoregulatory and anti-inflammatory abilities.

Furthermore, the results indicated that both hyalomin-A1 and B1 significantly suppressed the LPS-induced activation of the JNK subgroup of the MAPK signaling pathway by blocking JNK phosphorylation and, consequently, led to a reduction in MCP-1, IFN-γ, and tumor necrosis factor-α genes. The *in vivo* experiments identified that these peptides could inhibit the hind paw’s inflammation in mice depending on the dose administered. These anti-inflammatory functions were significantly present after nine days of administration. At a dose of 5 mg/kg of body weight, the mice could recover to a normal state after 21 days of administration of hyalomin-A1 or B1 [141].

Ticks have another mechanism of escape from the host’s defenses related to the presence of evasins, small cysteine-rich binding proteins secreted in their saliva. To neutralize chemokines and their signaling, ticks, such as *Rhipicephalus sanguineus* (commonly called the brown dog tick, kennel tick, or pantropical dog tick), secrete evasins [142]. Evasin-1 (P0C8E7) inhibits cell recruitment of chemokines CCL3, CCL3L1, and CCL4-mediated chemotaxis in L1.2/CCR5 transfectants *in vivo* and *in vitro*. Besides, it also inhibited CCL3-induced granulocyte recruitment in mice. Evasin-3 (P0C8E8) inhibits neutrophil recruitment and reduces inflammation. Treatment with this peptide resulted in inhibiting total cell accumulation in the synovial cavity in a mouse-induced arthritis model. Inhibition of neutrophil infiltration in the knee joint reduced induced hypernociception, reduced production of TNF-α in the periarticular tissues, and inhibition of leukocyte adhesion [142]. The peptide derived from the N-terminal region of evasin-4 (P0C8E9), which had an affinity with the chemokine CCL5, inhibited the activity of CCL5 in monocyte migration assays. This result suggests that evasin-4 derivatives can serve as a starting point for developing anti-inflammatory drugs [142].

Tian et al. [143] investigated the immunosuppressive peptide amphiregulin from the tick *Amblyomma variegatum* (the tropical bont tick). This peptide is composed of 40 amino acid residues (HLHMHGNGATQVFKPRLVLKCPNAAQLIQPGKLQRQLLLQ). In rat splenocytes, amphiregulin exerted significant anti-inflammatory effects by inhibiting the secretion of TNF-α, IL-1, IL-8, and IFN-γ *in vitro*. Compared to LPS, these inflammatory mediators’ inhibition was significant in all tested peptide concentrations (2, 4, and 8 µg/mL). Amphiregulin showed substantial elimination of free radicals and antioxidant activities in specific concentrations (5, 10, and 20 µg/mL) *in vitro* and also significantly inhibited the paw inflammation induced by adjuvant mice *in vivo* [143].

### Chilopod peptides

*Centipede*

Centipedes are part of the subphylum Myriapoda (class Chilopoda). *Scolopendra subspinipes mutilans* (Chinese red-headed centipede) is a component of natural extract formulation widely used in traditional Chinese and Korean medicine to treat various conditions due to its anti-inflammatory, antimicrobial, and analgesic effects [144]. It is a stable extract of which studies report its neuroinflammatory activity and efficacy as a mitigating agent of inflammation in rheumatoid arthritis, as well as antitumor and immunostimulant [145,146]. From the venom of*Scolopendra subspinipes mutilans* (Chinese redhead), the formyl peptide receptor 2 (FPR2) peptide with a chemo-attractive property for FRP2 on the neutrophils’ surface was isolated. Results evidenced the therapeutic effects of this peptide on rheumatoid arthritis by inhibiting the release of pro-inflammatory cytokines and the recruitment of neutrophils in the joint [147]. Scolopendrasin IX, another peptide isolated from the same centipede species, can down-regulate the expression of pro-inflammatory mediators such as TNF-α and IL-6, also having therapeutic effects against rheumatoid arthritis. In mouse neutrophils, peptides from this centipede species’ venom have a high potential to control the inflammatory process due to their targeted effects. However, the mechanism of action has not been clarified yet [147].

## Discussion

### Peptides and antitumor activities

When there is a failure in the inflammatory process’s control mechanism, the condition can evolve into chronic inflammation with consequent mutation and cell proliferation, thus creating an environment conducive to cancer development. In this context, numerous treatments rely on antineoplastic therapy, including chemotherapy, radiotherapy, and immunotherapy [148]. These therapeutic options can cause serious side effects and increase resistance to neoplastic cells, therefore continuous research intent to find new therapeutical options. Animal venoms have become an object of interest because they have specific and structurally stable components that can interact with and modulate their molecular targets, making them good therapeutic candidates [149].

Among the drugable candidates, peptides from different arthropod species can potentially control inflammatory processes and control malignant neoplasms [150]. For instance, among the various ant toxins, solenopsin A (derived from red imported fire ant- *Solenopsis invicta*) is a potent anti-angiogenic agent that inhibits the phosphorylation of Akt-1 and FOXO1a, a substrate of Akt, thus modulating the Akt signal transduction, phosphatidylinositol-3-kinase in mouse embryos (3T3-L1 and NIH3T3) and zebrafish [151]. In cell cultures of HepG2, MCF-7, and LoVo lines, this peptide proved to be an anti-angiogenic toxin that can reduce the levels of cytokines such as interleukin (IL) -1β, IL-6, IL-8, and NF-κB) [152]. Table 2 summarizes information regarding some venom peptides with antitumoral and anti-inflammatory activity.

**Table 2.** Examples of peptides from the Uniprot database with antineoplastic activities.

Animal (Source)	Peptide	Access number	Antitumoral activity	Ref.
**Insect**				
*Solenopsis invicta*	Solenopsin	-	Inhibits PIK3 activation, Akt and FOXO1 phosphorylation	[150-152]
*Apis mellifera*	Melitin	P01501	Activation of caspases, metalloproteinases and PLA2	[155-157]
	Phospholipase	P00630	Epidermal growth factor receptor (EGFr) reduction	[158]
	Bee venom	-	Reduction of Bcl-2 expression	[159,160]
*Polybia paulista*	Mastoparan 1	P0C1Q4	Induces mitochondrial permeability and cytochrome release	[161]
	Polybia MPI	-	Cytotoxicity against leukemic T lymphocytes	
**Arachnid**				
*Macrothele raven*	Macrothele raven venom	-	Antitumoral activity	[163,164]
*Haplopelma haunanum*	*Haplopelma haunanum* venom	-	Reduced cell growth and stimulation of the production of caspase 3 and 9	[167]
**Crustacean**				
*Buthus matensii karsch*	*Buthus matensii karsch* venom	-	Induce apoptosis by producing caspase 3 and down-regulating Bcl-2	[168]
*Androctonus mauritanicus* e *Androctonus australis*	Gonearrestide	-	Inhibition of cyclin-dependent kinase 4 (CDK4) and increased cell expression of cycle regulators and inhibitors (cyclin D3, p27, and p21)	[169]
*Leiurus quinquestriatus*	Chlorotoxin	P45639	Can bind endogenously to MMP-2 expressed in glioma cells	[170,171]
*Parabuthus schlechteri*	PBITx1	P60271	Selective toxin of the Na^+^ channel	[172]
**Chilopod**				
*Parafontaria laminata*			Suppressive activity of the focal adhesion kinase pathway (FAK) and the kinase pathway regulated by the extracellular signal (ERK)	[153-154]

In this line, the centipede glycosphingolipid peptide-7 from the millipede - *Parafontaria laminata armigera* exerts an antiproliferative effect on neoplastic cells and inhibits the focal adhesion kinase (FAK) pathway in addition to the signal-regulated kinase (Erk) 1 and 2, both involved in the proliferation of melanoma cells. This same peptide reduced proteins’ expression related to oral squamous cell carcinoma (cyclin D1) [153]. Regarding bee venom, melittin (*Apis mellifera*) is undoubtedly one of the most multifunctional toxins. In the fight against neoplastic cells, melittin can bind calmodulin and prevent cell proliferation, inducing the death of neoplastic cells through the activation of caspases and metalloproteinases (MMPs) [154,155]. In cells transformed by an oncogene, melittin activates PLA2, which destroys cancer cells and comprises another mechanism that acts as an antineoplastic agent. Through the PLA2-dependent mechanism of activation, melittin is effective in leukemic cell lines that are even resistant to TNF-α [156, 157].

PLA2 (*Apis mellifera*) is a toxin that negatively regulates transduction pathways related to cell survival and tumor invasion. Moreover, treatment with this peptide decreased epidermal growth factor (EGFr) [158]. BV is efficient in killing K1735M2 and B16 melanoma cells, halting the cell cycle at the G1 stage and, therefore, inhibiting cancer cells’ proliferation in a dose-dependent manner. Furthermore, BV treatment stimulated Bax production, a pro-apoptotic protein, and reduced the expression of Bcl-2, resulting in the formation of dimers with Bax and the consequent cell death [159, 160].

Mastoparan is a peptide isolated from wasp *Polybia paulista*, which alone can induce mitochondrial permeability; however, it does not have specificity in malignant cells. Though, when encapsulated in a liposome, this peptide could release cytochrome in human chronic myeloid leukemia cells [161]. Isolated from *Polybia paulista*, the Polybia MPI peptide has cytotoxicity against leukemic T lymphocytes, in addition to being able to reach the cells of the lipid membranes creating channels that provoke ionic permeabilization, depolarization, and consequent cell death [162].

Although spiders are a widespread species within the arthropod group, toxins that act as antineoplastic agents are understudied. Research has shown that the crude venom from *Macrothele raven* (Araneae, Hexathelidae) can arrest cancer cells via caspase 3 in treated cells, leading to the HeLa cell’s cell death. In breast cancer cells, the crude venom of this species caused cell death, in addition to causing a cell arrest in the G2/M and G0/ G1 cycles [163, 164].

The toxins obtained from the Chinese bird spider *Haplopelma hainanum* showed antitumor activity in a liver cancer cell line, decreasing cell growth, mitochondrial membrane potential, in addition to stimulating the production of caspase 3 and 9 and inducing apoptosis through a dependent mitochondrial pathway [165].

Scorpion venoms have been a promising target in cancer treatment, the most interesting being the long-chain toxins that act on K +, Cl-, and ion channels. For example, human breast cancer MCF-7 cells treated with *Buthus matensii karsch* toxin extract could induce apoptosis by producing caspase 3 and down-regulating Bcl-2. In *in vitro* studies, gonoearrestide, a peptide found in the fat-tailed scorpion *Androctonus mauritanicus* and *A. australis*, was able to kill neoplastic cells by arresting the cell cycle in the G1 phase due to inhibition of cyclin-dependent kinase 4 (CDK4) and increased cell expression of cycle regulators and inhibitors cyclin D3, p27, and p21 [166]. Also, this species' venom was able to block the cell cycle from the G0/G1 phase to the S phase [167].

Chlorotoxin (Cltx) is found in the venom of the Palestine yellow scorpion *Leiurus quinquestriatus*. *In vitro* studies showed that Cltx binds to glioma cells without affecting normal cells; Cltx can bind endogenously to MMP-2 expressed in glioma cells, thus generating a loss of the gelatinase activity of the glioma and decreasing the expression of MMP2. PBITx1, extracted from the burrowing thick tail scorpion *Parabuthus schlechteri*, is a selective toxin of the Na+ channel and structurally similar to Cltx, suggesting that it could act on chloride channels and arrest cancer cells [168-170]. This synthesized peptide showed low toxicity in clinical trials, inhibiting angiogenesis, a possible candidate to combat gliomas [171].

### Work limitations

Arthropods comprise a large phylum of invertebrate animals, and their particular biological and ecological characteristics vary according to each species. It is worth mentioning that numerous species have bioactive peptides in their venoms with anti-inflammatory activity, as observed in studies conducted *in vivo* and *in vitro*. Thus, we selected certain arthropods groups that provided more publications related to the theme when inquiring databases. We expected the present review to glimpse the theme and attract the audience's attention to this exciting research topic. A limitation of the study is about some elusive mechanisms of action of venom peptides reported by different laboratories that can be further explored for peptide drug development. Despite this, a handful of information allowed describing the peptides' significant “anti-inflammatory effects” from venom components of numerous arthropod species.

## Conclusion

Considerable diversity of bioactive molecules under investigation can be developed as therapeutic agents to treat numerous human diseases. Various research groups have studied different peptides identified in arthropod venoms to unravel their potential as anti-inflammatory agents. The selected examples listed herein comprise peptides found in the venom and hemolymph of diverse species of arthropods. Included in this review were arthropods related to insects (ants, bees, and wasps), crustaceans (shrimp and crabs), arachnids (scorpions and spiders), and chilopods (centipedes), all of them containing in their venom peptides with important anti-inflammatory activity. Peptides derived from arthropod venoms act on different inflammatory pathways, reducing pro-inflammatory cytokines both in *in vitro* and *in vivo* models. It is known that inflammation at an advanced stage can trigger malignant neoplasms and contribute to their exacerbation. Thus, multifunctional venom peptides that act on inflammatory pathways and pathways related to cancer deserve considerable attention in the present and future natural drug development programs. Consequently, arthropod venom peptides, which evolved over millions of years, comprise a rich source for discovering and developing peptides with potent pharmacological efficacy to treat inflammatory and malignant diseases. The disclosure of their specific mechanisms of action and application potential as therapeutic agents should continue in the years to come.

### Abbreviations

ALF: anti-lipopolysaccharide factor; ALS: amyotrophic lateral sclerosis; BAX: BCL2-associated X protein; BCL: B-cell lymphoma; BV: bee venom therapy; CAT: catalase; CD: cluster of differentiation; CMO: scorpion oligopeptides; COX: cyclooxygenase; DBPs: disulfide-bridged peptides; DPPH: 2,2-diphenyl-1-picrylhydrazyl; ERK: extracellular signal-regulated kinase; FAZ: cell surface death receptor; FDA: U.S. Food and Drug Administration; FPR-2: formyl peptide receptor-2; ICAM-1: intercellular adhesion molecule 1; IFN- γ: interferon gama; IL-1: interleukin 1; IL-1β: interleukin beta; IL-4: interleukin 4; IL-6: interleukin 6; IL-8: interleukin 8; IL-10: interleukin 10; IL-13: interleukin 13; iNOS: nitric oxide-induced synthase; JNK: c-Jun N-terminal kinases; LALF: limulus anti-lipopolysaccharide factor; LPS-BD: lipopolysaccharide-binding domain; LPS: anti-lipopolysaccharide; M-ALF: marsupenaeus anti-lipopolysaccharide factor; MAP: mitogen-activated protein; MAPK: mitogen-activated protein kinase; MHC-II: major histocompatibility complex 2; MPC: monocyte chemoactive protein; MPO: myeloperoxidase; NDBPs: non-disulfide-bridged peptides; NF-kβ: nuclear factor kappa beta; PAM: antimicrobial peptides; PAMP: pathogen-associated molecular pattern; PRISMA: preferred reporting items for systematic reviews and meta-analysis; PGE: prostaglandin; PLA2: phospholipase A2; PtALF: portunus trituberculatus anti-lipopolysaccharide factor; SALF: shrimp anti-lipopolysaccharide factor; SOD: superoxide dismutase; TGF-β1: transforming growth factor-β1; TLR: toll-like receptor; TNF- α: nuclear transcription factor-alpha; TRAP1: transient receptor potential ankyrin; VCAM: vascular adhesion molecule; β-GPB: guanine nucleotide-binding protein subunit beta. 
